# The epidemiology and aetiology of diarrhoeal disease in infancy in southern Vietnam: a birth cohort study

**DOI:** 10.1016/j.ijid.2015.03.013

**Published:** 2015-06

**Authors:** Katherine L. Anders, Corinne N. Thompson, Nguyen Thi Van Thuy, Nguyen Minh Nguyet, Le Thi Phuong Tu, Tran Thi Ngoc Dung, Voong Vinh Phat, Nguyen Thi Hong Van, Nguyen Trong Hieu, Nguyen Thi Hong Tham, Phan Thi Thanh Ha, Le Bich Lien, Nguyen Van Vinh Chau, Stephen Baker, Cameron P. Simmons

**Affiliations:** a Oxford University Clinical Research Unit, Wellcome Trust Major Overseas Programme, Hospital for Tropical Diseases, 764 Vo Van Kiet, District 5, Ho Chi Minh City, Vietnam; b Centre for Tropical Medicine, Nuffield Department of Clinical Medicine, Oxford University, Oxford, UK; c Department of Epidemiology and Preventive Medicine, Monash University, Melbourne, Australia; d The London School of Hygiene and Tropical Medicine, London, UK; e The Hospital for Tropical Diseases, Ho Chi Minh City, Vietnam; f Hung Vuong Hospital, Ho Chi Minh City, Vietnam; g Dong Thap Hospital, Cao Lanh, Vietnam; h District 8 Hospital, Ho Chi Minh City, Vietnam; i Children's Hospital No. 1, Ho Chi Minh City, Vietnam; j Department of Microbiology and Immunology, University of Melbourne, Victoria, Australia

**Keywords:** Diarrhoeal disease, Infectious diarrhoea, Infants, Epidemiology, Cohort study, Rotavirus

## Abstract

**Objectives:**

Previous studies indicate a high burden of diarrhoeal disease in Vietnamese children, however longitudinal community-based data on burden and aetiology are limited. The findings from a large, prospective cohort study of diarrhoeal disease in infants in southern Vietnam are presented herein.

**Methods:**

Infants were enrolled at birth in urban Ho Chi Minh City and a semi-rural district in southern Vietnam, and followed for 12 months (*n* = 6706). Diarrhoeal illness episodes were identified through clinic-based passive surveillance, hospital admissions, and self-reports.

**Results:**

The minimum incidence of diarrhoeal illness in the first year of life was 271/1000 infant-years of observation for the whole cohort. Rotavirus was the most commonly detected pathogen (50% of positive samples), followed by norovirus (24%), Campylobacter (20%), Salmonella (18%), and Shigella (16%). Repeat infections were identified in 9% of infants infected with rotavirus, norovirus, Shigella, or Campylobacter, and 13% of those with Salmonella infections.

**Conclusions:**

The minimum incidence of diarrhoeal disease in infants in both urban and semi-rural settings in southern Vietnam was quantified prospectively. A large proportion of laboratory-diagnosed disease was caused by rotavirus and norovirus. These data highlight the unmet need for a rotavirus vaccine in Vietnam and provide evidence of the previously unrecognized burden of norovirus in infants.

## Highlights

• The diarrhoeal disease burden in a large, prospective infant cohort in Vietnam is defined.
• Minimum incidence of clinic-based diarrhoea in infants: 271/1000 infant-years.
• Rotavirus was most commonly identified, followed by norovirus and bacterial pathogens.
• Frequent repeat infections with the same pathogen within 1 year.
• Inclusion of rotavirus in the immunization schedule for Vietnam is warranted.

## 1 Introduction

Diarrhoea remains a substantial cause of morbidity and mortality amongst children globally.^1,2^ In a study in rural central Vietnam, the incidence of diarrhoea in children under 5 years of age was found to exceed 115 episodes/1000 child-years.^3^ Risk factors for diarrhoea in Vietnam include, as in many settings, male gender, age less than 2 years, and poor socioeconomic indicators such as household crowding and poor hygiene habits.^4,5^ There are no equivalent population-based estimates of diarrhoea in southern tropical Vietnam, where approximately 40% of the country's population live.

A recent study in southern Vietnam illustrated the relative contributions of rotavirus, norovirus, and the bacterial pathogens *Shigella spp*, *Salmonella spp*, and *Campylobacter spp* as the aetiological agents of diarrhoea in hospitalized children under 5 years of age in Ho Chi Minh City (HCMC).^6^ How well these data represent the community level burden of diarrhoeal disease is unclear. Further, these data suggest that the majority of hospitalized diarrhoea cases are in children <12 months of age,^6^ which is the pivotal age group at which rotavirus vaccine should be targeted. Longitudinal community cohort studies provide an opportunity to evaluate the epidemiology and disease burden of diarrhoea to a fuller scale than hospital-based research. However, few studies have evaluated the incidence of diarrhoea in Vietnam,^3,7^ and to date none have focused exclusively on the tropical south of the country. To address this knowledge gap, we sought to define the burden, aetiology, and risk factors for diarrhoeal disease through community cohorts of infants in two distinct settings in this densely populated, rapidly industrializing region. A better understanding of the epidemiology and aetiologies of diarrhoeal disease in southern Vietnam will inform rational public health interventions.

## 2 Methods

### 2.1 Description of the cohort

The cohort structure and methodology have been described previously.^8^ Briefly, pregnant women were enrolled from 2009 to 2013 in southern Vietnam in two locations: women resident in central HCMC, the largest city in southern Vietnam, were enrolled at Hung Vuong Obstetric Hospital in HCMC; women resident in Cao Lanh District, Dong Thap Province, which is 120 km southwest of HCMC and situated in a semi-rural setting, were enrolled at Dong Thap Provincial Hospital. After delivery, infants were enrolled and followed up for the first 12 months of life with routine visits at 2, 4, 6, 9, and 12 months of age. A brief questionnaire detailing growth and illness in the preceding period since the last visit was administered, and a series of samples (blood, throat swab, nasopharyngeal swab) was collected at each routine visit.

### 2.2 Diarrhoeal episode detection

During the 12 months of follow-up, passive detection of diarrhoeal illness was performed, in which families were asked to take their child to a designated study clinic if the infant was unwell. At presentation, a brief clinical report was collected, as well as a stool sample. If the child was admitted, a detailed clinical evaluation was recorded. Blood samples were collected at the discretion of the treating physician. A new episode of diarrhoea was defined by ≥7 days between the onset dates of symptoms. Diarrhoea was defined as three watery loose stools or at least one bloody/mucoid diarrhoeal stool within 24 h,^9^ or an increase in stool frequency as determined by the parent's judgement.

A secondary source of data on diarrhoeal episodes were self-reports by the mother of diarrhoeal illness in their infant for the period prior to each study visit.

### 2.3 Laboratory analysis

Stool samples collected from diarrhoeal episodes were stored at 4 °C until transport within 24 h and were then stored at −80 °C until further testing. One-step reverse transcriptase (RT) PCRs for rotavirus and norovirus genogroups I and II (GI and GII) were performed using RNA Master Hydrolysis Probes (Roche Applied Sciences, UK) on a LightCycler 480 (Roche Applied Sciences, UK) with the primers and probe sequences and PCR cycling conditions described previously.^10^ Real-time PCR cycling conditions for Shigella (target *ipa*H) and Campylobacter (*Campylobacter jejuni* target: *hip*O; *Campylobacter coli* target: *gly*A) were as follows: 95 °C for 15 min, followed by 40 cycles of 95 °C for 5 s, 60 °C for 30 s, 72 °C for 30 s, as described previously.^11,12^ Salmonella was detected using an in-house assay targeting the *inv*A gene, which is conserved across the eight *Salmonella* subspecies, with cycling conditions as follows: 95 °C for 15 min, followed by 45 cycles of 95 °C for 5 s, 60 °C for 60 s. The sequences of the primers and probe for the *inv*A gene were as follows: forward 5′-TCATCGCACCGTCAAARGA-3′, reverse 5′-CGATTTGAARGCCGGTATTATT-3′, probe: 5′-FAM-ACGCTTCGCCGTTCRCGYGC-BHQ1-3′. The limit of detection was 5 copies/reaction. Stool samples were not available from self-reported diarrhoea episodes.

### 2.4 Statistical analyses

Two separate incidence measurements were calculated: one evaluating diarrhoeal presentations at a study clinic and/or admitted to hospital, and the other based solely on self-reported diarrhoeal illness derived from information collected at the routine follow-up visits. These data were not merged. Infant-years of observation (IYO) for each infant were derived from the date of birth and date of exit from the study due to either completion of follow-up, documented early withdrawal, or loss to follow-up, defined by the last routine visit or illness presentation, whichever was later, if the full 12-month follow-up period was not completed. Pathogen-specific incidence estimates were not calculated due to low counts, but the incidence of aetiological groups (bacterial, viral, or mixed infection) was evaluated. Comparisons between groups were made using the Kruskal–Wallis test for continuous variables with non-normal distributions and the Chi-square test for categorical variables.

Multivariable negative binomial regression was used to identify risk factors associated with severe diarrhoea presenting to a study clinic and/or admitted to hospital. Regression was performed independently for each study site due to the heterogeneity in risk profiles between HCMC and Dong Thap. Factors were included in the multivariable model according to hypothesized associations determined a priori (maternal characteristics, socioeconomic indicators, household elevation), as well as those found to be significantly associated in the univariable analysis (*p* < 0.05). All analyses were performed in Stata v. 13 (StataCorp, College Station, TX, USA).

### 2.5 Spatial clustering analyses

To investigate the presence of spatial clustering of diarrhoeal illness, we used a Bernoulli model with all diagnosed episodes of diarrhoea as cases, and children without any reported history of diarrhoeal episodes as the background population using SaTScn v. 9.1.1 (http://www.satscan.org/). Each pathogen in turn was also considered as a case, with the control group remaining all children in the cohort with no reported episode. For the analyses, the upper limit for cluster detection was specified as 50% of the study population. The significance of the detected clusters was assessed by a likelihood ratio test, with a *p*-value obtained by 999 Monte Carlo simulations generated under the null hypothesis of a random spatiotemporal distribution.

### 2.6 Ethics

Four hospitals in HCMC (Hospital for Tropical Diseases, Hung Vuong Obstetric Hospital, District 8 Hospital, Children's Hospital 1) and Dong Thap Provincial Hospital participated in the study. The protocol was approved by the institutional review boards of all these hospitals, as well as the Oxford Tropical Research Ethics Committee. Written informed consent was obtained from all participants.

## 3 Results

### 3.1 Baseline characteristics of the cohorts

From July 2009 to December 2013, a total of 6706 infants were enrolled in the birth cohort from 6679 mothers (27 sets of twins). A total of 6239.4 infant-years of observation (IYO) were recorded for these children. In Dong Thap, there were 2458 infants enrolled with 2199.4 IYO, and in HCMC there were 4248 infants enrolled with 4040 IYO. The full 12-month follow-up was completed by 87% of the cohort, with 33% (289/884) of early exits occurring after at least 9 months of cohort membership. Slightly over half of enrolled babies were male (52%), with roughly 5% being of low birth weight (<2500 g) (Table 1). The majority of children (91%) were breastfed after birth; 33% were exclusively breastfed. The use of milk formula after birth was more frequently reported in HCMC (92%) compared with Dong Thap (26%). Households in Dong Thap were more likely to have characteristics of lower socioeconomic status compared to HCMC, with a higher prevalence of household crowding, a lack of flush toilets, use of river water as the primary water source, and lower maternal education level (Table 1).

### 3.2 Incidence of diarrhoeal disease

During the follow-up period there were 1690 diarrhoeal presentations detected through clinic-based surveillance. The majority of these illnesses were treated on an outpatient basis (91.4%). The minimum incidence of diarrhoeal presentations estimated for the cohort as a whole was 271/1000 IYO. In Dong Thap, this figure was 604.3/1000 IYO and in HCMC was 89.4/1000 IYO. The minimum incidence estimates for hospitalized diarrhoeal illness in each location were 57.3/1000 IYO and 4.5/1000 IYO, respectively. There were 1656 self-reported diarrhoeal episodes at routine follow-up visits, corresponding to an incidence of 265.4/1000 IYO for the entire cohort. The incidence of self-reported diarrhoea was similar between the study sites: in Dong Thap it was 318.3/1000 IYO and in HCMC it was 236.6/1000 IYO.

### 3.3 The aetiology of diarrhoeal disease

Of the 1690 unique diarrhoeal presentations, 1309 (77%) had a corresponding stool sample. Of these, a total of 748 (57%) tested positive for one or more of the six pathogens screened. Among the positive samples, rotavirus was the most commonly detected pathogen (53%, 395/748); notably almost a third of these samples were also positive for another pathogen (32%, 128/395). Norovirus was identified in 24% (176/748) of positive samples, of which 88 (50%) samples were also positive for another pathogen. PCR amplifications consistent with the presence of Shigella, Salmonella, and Campylobacter were identified in 16% (*n* = 117), 18% (*n* = 135), and 20% (*n* = 152) of samples, respectively. Amongst the norovirus infections, GII was predominant (167/176, 95%), with an additional two samples positive for both GI and GII. Of the Campylobacter infections, *C. jejuni* was detected most often (94%, 143/152), followed by *C. coli* (6%). Mixed infections accounted for 26% (192/748) of all positive samples, most of which (68%, 130/192) were a mixed viral/bacterial infection.

**Figure 1:**
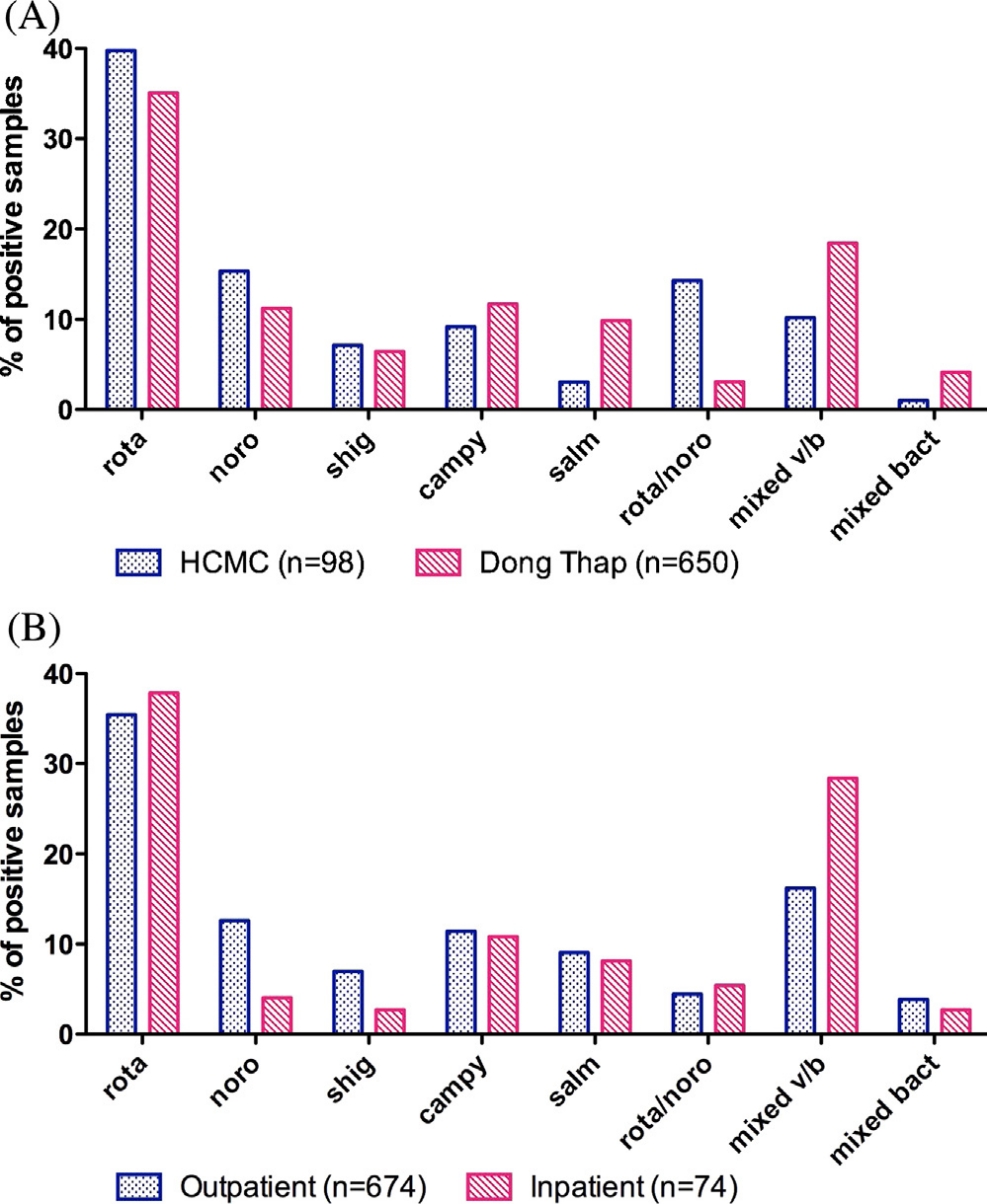
Aetiology of diarrhoeal disease. The proportion of diarrhoeal episodes (among those with a positive stool sample) that were positive for each single pathogen or combination of pathogens, (A) by study site, and (B) by hospitalization status.

Stool samples were collected from a far greater proportion of diarrhoeal episodes in the Dong Thap cohort compared to the HCMC cohort (86% vs. 47%). For inpatient diarrhoeal episodes in particular, the completeness of stool sample collection was far higher in Dong Thap (103/126; 82%) than in HCMC (5/18; 28%). This was due to difficulties in identifying hospital admissions of cohort members in real time in HCMC. The proportion of samples positive for at least one pathogen did not differ between sites, but was collectively higher among inpatient samples than outpatient samples (69% vs. 56%). The distribution of aetiologies differed significantly between HCMC and Dong Thap (Chi-square *p* < 0.001), with viral infections more common in HCMC and bacterial and mixed viral/bacterial infections more common in Dong Thap (Figure 1A). Mixed viral/bacterial infections were more common among hospitalized diarrhoeal cases than outpatients, however the overall distribution of aetiologies was not significantly different between outpatients and inpatients (Chi-square *p* = 0.09; Figure 1B). Among all detected diarrhoeal episodes, infections with a mixed viral/bacterial aetiology were most likely to be admitted to hospital (26%, 35/133), followed by viral infections (17%, 67/391).

**Figure 2:**
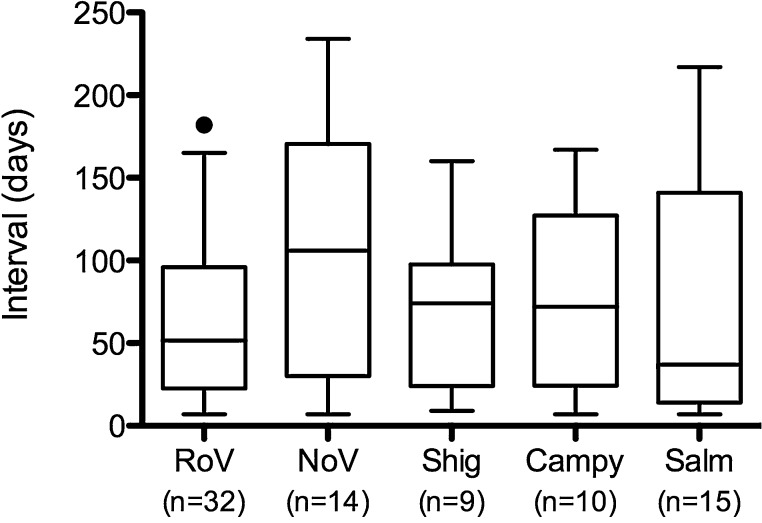
Time interval between repeated infections with enteric pathogens. Each box-and-whiskers plot shows the distribution of time intervals (in days) between repeated infections with the same enteric pathogen. Boxes indicate the median and interquartile range, and the whiskers indicate the 5^th^ and 95^th^ percentiles. RoV, rotavirus; NoV, norovirus; Shig, Shigella; Campy, Campylobacter; Salm, Salmonella. The numbers below each pathogen label indicate the total number of secondary or tertiary infections for that pathogen.

Repeat infections with the same pathogen were identified in a subset of infants. Rotavirus was identified in 365 infants, 32 of whom (9%) had at least two discrete rotavirus infections separated by at least 7 days. This proportion was the same for norovirus (15/163), Shigella (10/108), and Campylobacter (12/141). Of the 120 infants with Salmonella infection, 15 (13%) had at least two distinct episodes where Salmonella was detected. Figure 2 shows the distribution of the interval between repeated infections, by pathogen. The median interval between repeated infections ranged from 37 days for Salmonella to 106 days for norovirus, but this difference was not statistically significant (Kruskal–Wallis *p* = 0.44).

### 3.4 Clinical characteristics by aetiological group

Amongst all 1690 diarrhoeal episodes detected by clinic-based surveillance, the median age of the affected infants was 6.5 months (interquartile range (IQR) 4.6–8.7 months). A total of 55% (*n* = 934) of all diarrhoeal cases were male. Amongst episodes with an identified aetiology, infants with mixed infections tended to be slightly older (median 8 months) compared to those with the other aetiological groups (Table 2 ). The median axillary temperature at hospital admission was 37.8 °C (IQR 37–38.5 °C), which did not differ significantly between aetiological groups. Infants admitted to hospital with mixed viral/bacterial infections had a higher proportion of neutrophils (median 54%, IQR 36–59%; Kruskal–Wallis *p* = 0.002) and lower proportion of lymphocytes (median 35.3%, IQR 22.6–48.2%; Kruskal–Wallis *p* = 0.004) than those in the other aetiological groups, including those with negative samples. Infants admitted with bacterial infections were most likely to be prescribed an antimicrobial (86%, 19/22) (*p* = 0.043, Chi-square). The average length of stay in hospital for all admitted diarrhoeal episodes was 5 days (IQR 3–7 days).

### 3.5 Risk factors for diarrhoeal disease

Risk factors for diarrhoea were investigated by site. In the unadjusted analysis, increased maternal education was protective against diarrhoea in HCMC, whereas male sex, household crowding, use of a piped water supply, and filtering drinking water were all significant risks (Table 3). In a multivariable analysis, maternal education (incidence rate ratio (IRR) 0.75, 95% confidence interval (CI) 0.56–1.00) remained independently associated with protection, and household crowding (≥2 people/room; IRR 1.45, 95% CI 1.07–1.95) along with filtering drinking water (IRR 1.81, 95% CI 1.17–2.81) remained risk factors in this setting.

In Dong Thap, the most important protective factors included maternal age at delivery, maternal education, and filtering of the drinking water supply (Table 4). Male sex and the lack of a flush toilet were risk factors in this setting. After adjusting for confounding, male sex remained the only strongly associated risk factor (IRR 1.20, 95% CI 1.04–1.40), and maternal age (IRR 0.98, 95% CI 0.96–0.99) and education (IRR 0.75, 95% CI 0.62–0.91) remained protective.

### 3.6 Spatial clustering

**Figure 3:**
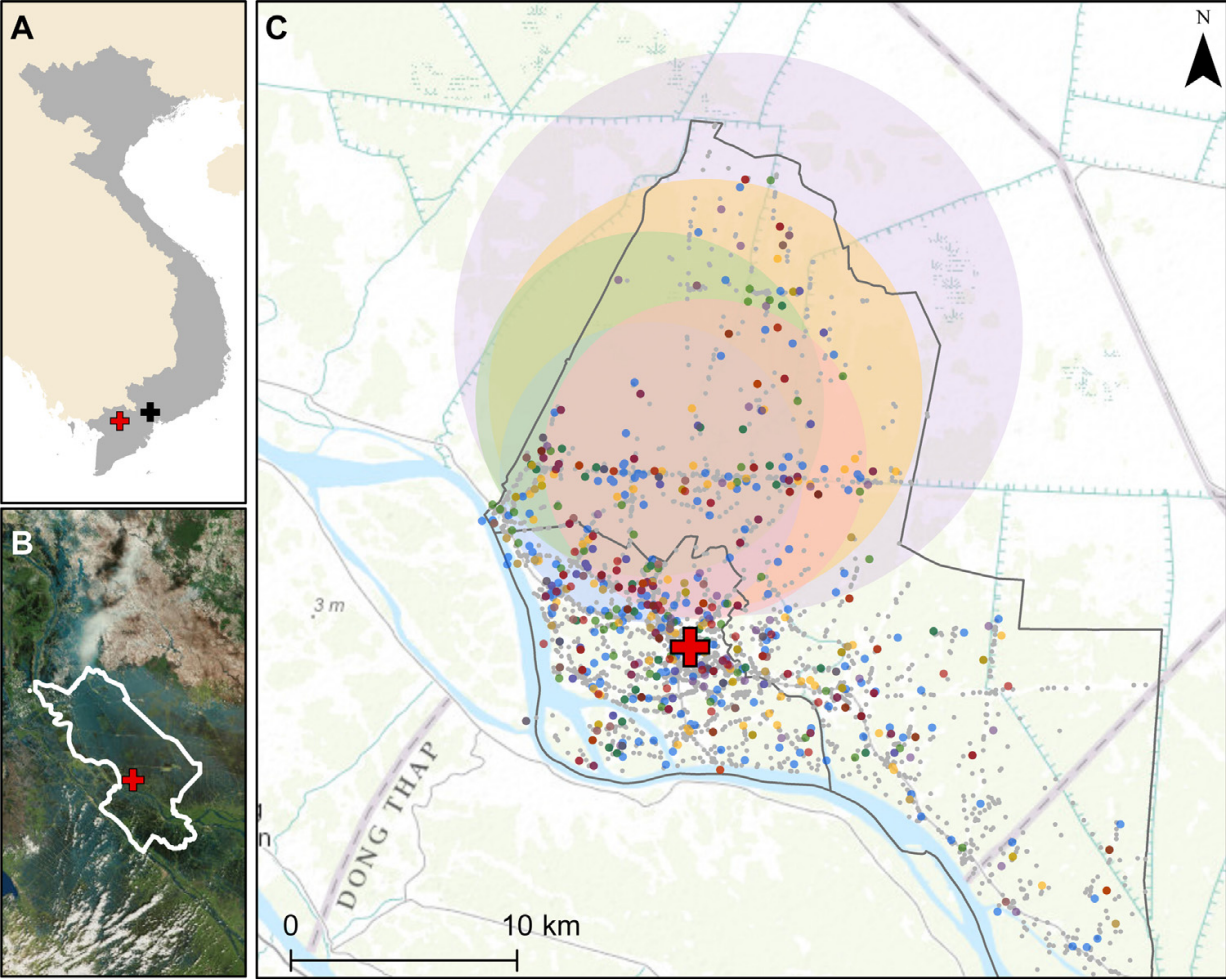
Spatial clustering of diarrhoeal cases in Dong Thap Province. (A) Vietnam is shown in grey, with Ho Chi Minh City (HCMC) indicated with the black cross and Dong Thap indicated with the red cross. (B) Location of Dong Thap Province, with the hospital indicated by the red cross. (C) Locations of households with diarrhoea shown with coloured points, as follows: blue, rotavirus; green, norovirus; red, Shigella; orange, Salmonella; purple, Campylobacter. Grey points show the locations of the households of children in the cohort for whom diarrhoea was not reported during the period of follow-up. Shaded circles indicate the location of the significant clusters, with colours corresponding to the individual pathogens listed above. The red cross shows the location of Dong Thap Provincial Hospital. Grey borders show the borders of Cao Lanh district (large border) and Cao Lanh town (small, embedded border). Actual household locations have been jittered for display on the map.

As shown in Figure 3, in Dong Thap there was evidence of spatial clustering for each detected pathogen. For all-cause diarrhoea, a cluster was identified with a radius of 6.7 km in the northwest region of the study area (relative risk (RR) 1.79, *p* < 0.001). All of the pathogen-specific clusters centred generally around the same area, in the more rural part of the Dong Thap study area, with radii ranging from 6.6 km (rotavirus) to 12.4 km (Campylobacter) and RRs from 2.3 (rotavirus) to 3.7 (Shigella). No significant spatial clustering was identified in HCMC (data not shown).

## 4 Discussion

Diarrhoea remains one of the most common yet preventable conditions affecting the poorest children globally.^1^ Through a large, longitudinal birth cohort, a substantial burden of diarrhoeal disease in the first year of life was identified in southern Vietnam, with an estimated minimum incidence of 271/1000 IYO. This is an order of magnitude less than an estimate in infants aged <12 months from the late 1990s in rural Hanoi (3.3/child/year),^7^ yet it is higher than the incidence estimated in children under 5 years of age in central Vietnam in 2001–2003 (115/1000 child-years).^3^ Differences in disease incidence may have arisen from study design, as the study from rural Hanoi included partially active surveillance. Furthermore, although Dong Thap seemingly had a much higher minimum incidence (604/1000 IYO) than HCMC (89/1000 IYO), the large difference is very likely due to under-ascertainment in HCMC, as the number of healthcare providers in this urban setting is much greater than in semi-rural Dong Thap,^13^ and cohort participants therefore had greater opportunity to seek care at non-study clinics.

Viral infections represented the largest burden amongst all diagnosed diarrhoeal presentations in this study, confirming an earlier hospital-based study in HCMC.^6^ The distribution of aetiologies between the two sites was comparable, with mixed viral infections identified more frequently in HCMC. This may be confounded by under-ascertainment of hospitalized cases, in particular in HCMC, since hospitalized cases were more likely to be bacterial. Campylobacter was the most frequently detected bacterial pathogen in our cohort, which is in contrast to the recently published Global Enteric Multicenter Study (GEMS), which identified Shigella as the third most common cause of disease, behind rotavirus and Cryptosporidium, in moderate to severe diarrhoea in the first year of life across seven different Asian and African countries.^2^ As no control specimens were collected from healthy children in the present study, the aetiological role of the detected organisms cannot be determined. However, results from a hospital-based study in HCMC suggest that these organisms are not frequently identified in children without diarrhoea, with only 13% of approximately 600 non-diarrhoeal controls positive for an enteric pathogen.^6^

Through this work a large burden of potentially vaccine-preventable rotavirus disease was identified in infants. Over half of all samples with an identified aetiology were positive for rotavirus, with 13% of all rotavirus episodes admitted to hospital. Rotavirus vaccine is available as a ‘user pays’ product in Vietnam (predominantly the Rotarix monovalent vaccine (GlaxoSmithKline)), but uptake is low due to the prohibitive cost (US$ 70–80) and a lack of vaccine availability in many regions, including Dong Thap. Only 24% of children in HCMC were vaccinated against rotavirus in our cohort. The Vietnamese Ministry of Health has sponsored a locally produced, live-attenuated monovalent rotavirus candidate vaccine, with some success in the early stages of clinical evaluation.^14^ Previous work has shown that rotavirus vaccination, if GAVI-subsidized, would be cost-effective in Vietnam,^15^ and safe when co-administered within the current expanded programme on immunization (EPI) structure.^16^ Furthermore, immune responses (IgA and serum neutralizing antibody) measured against the pentavalent vaccine (RotaTeq) in Vietnamese children were shown in one study to be comparable to those among children in Latin America and Europe.^17^ This suggests that rotavirus vaccination in Vietnam may not suffer from the same level of reduced immunogenicity that has been observed to occur with orally administered enteric vaccines in developing countries.^18^

The majority of the identified infections were found to have occurred after 6 months of age, potentially due to the waning of protective maternal antibody and generally high rates of breastfeeding after birth,^19,20^ and increased exposure to pathogens with the start of consumption of solid foods. The risk factors for diarrhoeal disease identified through this work, including household crowding, low maternal age, and male sex, are generally consistent with the literature.^4,5^ In HCMC, drinking filtered piped water was a significant risk for diarrhoeal disease, although the number of families reporting filtering was relatively low. This may be due to the use of ceramic filters that have pores too large to mechanically prevent viruses from entering the drinking water supply.^21^ The absence of a measurable protective effect of rotavirus vaccination in HCMC likely reflects the imperfect case ascertainment, as well as the fact that approximately 50% of diarrhoeal episodes with a known aetiology were associated with pathogens other than rotavirus. The identification of increased spatial risk for diarrhoeal disease in the north-western region of Cao Lanh District in Dong Thap may represent a hotspot of transmission, due potentially to poor sanitation or waste management practices.

The most important limitation in this work was the passive nature of diarrhoeal disease episode detection. Although the staff made every effort to ensure disease episodes were recorded, an unknown number of infants with diarrhoeal disease may have attended clinics other than ours, especially in HCMC, and it is acknowledged that the interpretation of the present results is dependent on this limitation. Therefore, the minimum incidence measurements herein likely underestimate the true burden, particularly in HCMC, and the risk factor and spatial analyses may be biased by misclassification of some infants with undetected diarrhoeal illness. This may also have affected the conclusions on diarrhoeal aetiology, if the distribution of pathogens among episodes from which no specimen was available differed from those specimens tested. The overall loss to follow-up rate was low, although such bias may also be present and important to consider. Finally, the number of pathogens screened for was limited and may explain the lack of an identified pathogen in almost half of the cases. In particular, screening was not performed for any viruses beyond norovirus and rotavirus, and parasites and diarrhoeagenic *Escherichia coli*, which are known to be prevalent amongst children with diarrhoea in industrializing countries, were not investigated.^2^

Further work to more fully determine the epidemiology of diarrhoeal disease in this setting is warranted, particularly in the face of emerging antimicrobial resistance.^6,22^ Active, community-based surveillance of high-risk populations would provide a more accurate estimation of the true extent of the burden. Furthermore, as roughly 40% of diarrhoeal episodes collected in the present cohort study lacked a final diagnosis, investigation into the prevalence of additional pathogens, particularly Cryptosporidium,^2^ would help local clinicians to better understand the range of potential aetiologies and corresponding therapies for their patient population. To explore these questions, enrolment into a cohort study of young children aged 1–5 years, as an extension of this birth cohort study, has recently been completed, which includes active surveillance for diarrhoeal disease and diagnosis of viral and bacterial gastrointestinal pathogens.^23^ Through this, it will also be possible to explore the relative pathogenicity of isolated organisms as well as distinguish reinfection from long-term carriage, due to the collection of stool from healthy children as well.

In conclusion, the most comprehensive epidemiological description of paediatric diarrhoea in infancy in southern Vietnam, to date, is presented herein. A high burden of diarrhoeal disease in infants under the age of 12 months in both an urban and semi-rural setting is documented, with a large proportion due to vaccine-preventable rotavirus infection. Future efforts to integrate either a GAVI-subsidized or a domestically produced rotavirus vaccine into the national EPI schedule should be pursued.

## Figures and Tables

**Table 1:** Baseline characteristics of Ho Chi Minh City (HCMC) and Dong Thap participants

Characteristic	HCMC	Dong Thap	Total
Total number of infants	4248	2458	6706
Infant years of follow-up	4040	2199	6239
Maternal			
Age at delivery, years	28 (24–32)	25 (22–29)	27 (23–31)
Education			
Lower secondary or below	2555 (60.2)	1894 (77.1)	4449 (66.4)
Higher secondary or above	1692 (39.8)	561 (22.9)	2253 (33.6)
Infant			
Male sex	2248 (52.9)	1255 (51.1)	3503 (52.2)
Low birth weight^a^	199 (4.7)	110 (4.5)	309 (4.6)
Rotavirus vaccine^b^	1035 (24.4)	0 (0)	1035 (15.4)
Breastfed after birth?			
Yes, exclusively	364 (8.6)	1823 (74.2)	2187 (32.6)
Yes, plus formula	3299 (77.7)	620 (25.2)	3919 (58.4)
No, only formula	585 (13.8)	15 (0.6)	600 (8.9)
Started food before 6 months	1715 (42.2)	238 (11.2)	1953 (31.6)
Older sibling	2476 (58.3)	1161 (47.3)	3637 (54.3)
Household			
Elevation ≤3 m	996 (23.4)	207 (8.4)	1203 (17.9)
Household crowding^c^	2727 (66.8)	1701 (77.4)	4428 (70.5)
Toilet type			
Own flush	3704 (87.2)	1394 (56.8)	5098 (76.1)
Shared flush	524 (12.3)	63 (2.6)	587 (8.8)
None/bush	1 (0)	599 (24.4)	600 (9.0)
Other	18 (0.4)	398 (16.2)	416 (6.2)
Water source			
Piped to home	2844 (67.0)	761 (31.0)	3605 (53.8)
Piped to public tap	51 (1.2)	277 (11.3)	328 (4.9)
Bottled	1292 (30.4)	190 (7.7)	1482 (22.1)
River/stream	1 (0)	1145 (46.7)	1146 (17.1)
Other	59 (1.4)	81 (3.3)	140 (2.1)
Water treatment			
Boil	2642 (62.2)	1662 (67.7)	4304 (64.2)
Filter	282 (6.6)	582 (23.7)	864 (12.9)
None	1311 (30.9)	193 (7.9)	1504 (22.4)
Other	12 (0.3)	17 (0.7)	29 (0.4)
Pigs in household	16 (0.4)	242 (9.9)	258 (3.8)
Poultry in household	134 (3.2)	761 (31.0)	895 (13.4)

a <2500 g.

b One or more doses.

c Two or more people per room.

**Table 2:** Incidence and age of all diarrhoeal presentations and clinical characteristics of inpatient admissions for diarrhoeal disease

Characteristic	Viral infections	Bacterial infections	Mixed viral/bacterial	Negative	*p*-Value^a^
All diarrhoeal presentations					
Age (months), median (IQR)	7.3 (5.6–9.1)	6.7 (4.6–9.2)	8.0 (6.2–9.4)	5.9 (4.0–7.8)	<0.001
Total count	391	227	133	563	
Total count, Dong Thap	322	207	123	492	
Total count, Ho Chi Minh	69	20	10	71	
Incidence per 1000 IYO	62.7	36.4	21.3	90.2	
Incidence per 1000 IYO, Dong Thap	146.4	94.1	55.9	223.7	
Incidence per 1000 IYO, Ho Chi Minh	17.1	5.0	2.5	17.6	
Inpatient admissions					
Total, *n* (%)	67 (17.1)	22 (9.7)	35 (26.3)	57 (10.1)	<0.001
Incidence per 1000 IYO	10.7	3.5	5.6	9.1	
Vital signs at day 1 of admission					
Maximum temperature, °C	37.5 (37.0–38.5)	37.9 (37.0–39)	37.8 (37.5–38.5)	37.8 (37.0–38.5)	0.748
Maximum heart rate, beats/min	130 (120–130)	127 (120–130)	120 (120–126)	120 (120–130)	0.049
Maximum respiratory rate, breaths/min	36 (32–40)	37 (34–40)	34 (30–38)	36 (32–40)	0.137
Vomiting day 1, *n* (%)	32 (47.8)	8 (36.4)	21 (60.0)	21 (36.8)	0.134
Haematology day 1, median (IQR)					
AST, U/l	78 (51–100)	30 (22–38)	65 (50–181)	89 (86–117)	0.139
ALT, U/l	65 (15–73)	36 (10–62)	36 (19–206)	38 (20–123)	0.729
WBC, ×10^9^/l	8.9 (6.6–11.4)	10.3 (7.64–13.7)	9.2 (7.3–13.0)	8.8 (5.6–10.9)	0.268
Neutrophils, %	49.3 (33.3–61.9)	43.0 (27.5–53.9)	53.7 (35.5–59.4)	38 (23.3–49.3)	0.002
Lymphocytes, %	36.3 (25.4–50.8)	38.5 (32.7–50.6)	35.3 (22.6–48.2)	49.5 (34.3–60.7)	0.004
Maximum HCT, %	35 (33–37)	34 (33–36)	37 (34–38)	35 (33–39)	0.094
Platelets, ×10^9^/l	338 (271–427)	296 (257–429)	375 (270–437)	319 (240–392)	0.426
Antimicrobial use, *n* (%)	35 (52.2)	19 (86.4)	20 (57.1)	34 (59.6)	0.043
If yes, duration (days), median (IQR)	5 (3–6)	5 (5–6)	5 (4–6)	5 (4–6)	0.624
Admission to ICU, *n* (%)	2 (3.0)	0 (0)	0 (0)	1 (1.8)	0.640
Parenteral fluid administered, *n* (%)	12 (19.4)	2 (9.1)	5 (14.3)	6 (10.5)	0.464
Length of hospital stay (days), median (IQR)	5 (3–6)	4 (3–5)	4 (3–6)	4 (3–6)	0.711

IQR, interquartile range; IYO, infant-years of observation; AST, aspartate aminotransferase; ALT, alanine aminotransferase; WBC, white blood cell count; HCT, haematocrit; ICU, intensive care unit

a *p*-Value from Chi-square test or Kruskal–Wallis test, as appropriate.

**Table 3:** Risk factors for diarrhoeal presentation in Ho Chi Minh City (HCMC)

Characteristic	IYO	Outpatient		Hospitalized		Total diarrhoea		Unadjusted IRR	Adjusted IRR	*p*-Value
		Cases	CI	Cases	CI	Cases	CI			
Infant-years of follow-up	4040	343	85	18	4	361	89			
Maternal										
Age at delivery	-	-	-	-	-	-	-	1.01 (0.98–1.03)	1.01 (0.98–1.03)	0.555
Education										
Lower secondary or below	2410.7	237	98	12	5	249	103	1.00	1.00	
Higher secondary or above	1629	106	65	6	4	112	69	0.67 (0.51–0.87)^d^	0.75 (0.56–1.00)^d^	0.053
Infant										
Sex										
Female	1895.6	140	74	7	4	147	78	1.00	1.00	
Male	2144.3	203	95	11	5	214	100	1.29 (1.00–1.66)^d^	1.24 (0.96–1.61)	0.105
Low birth weight^a^										
No	3853.5	334	87	16	4	350	91	1.00	1.00	
Yes	186.5	9	48	2	11	11	59	0.66 (0.33–1.31)	0.69 (0.34–1.40)	0.308
Rotavirus vaccine^b^										
No	3018.5	267	88	17	6	284	94	1.00	1.00	
Yes	1021.5	76	74	1	1	77	75	0.81 (0.6–1.09)	0.91 (0.66–1.24)	0.563
Breastfed after birth?										
Yes, exclusively	340.7	30	88	3	9	33	97	1.00	1.00	
Yes, plus formula	3138.7	267	85	11	4	278	89	0.92 (0.59–1.44)	0.87 (0.55–1.37)	0.541
No, only formula	560.6	46	82	4	7	50	89	0.92 (0.54–1.59)	0.87 (0.50–1.53)	0.637
Older sibling										
No	1699.3	130	77	7	4	137	81	1.00	1.00	
Yes	2340.4	213	91	11	5	224	96	1.19 (0.92–1.54)	1.03 (0.77–1.38)	0.838
Household										
Elevation ≤3 m										
No	3086.4	274	89	18	6	292	95	1.00	1.00	
Yes	953.5	69	72	0	0	69	72	0.77 (0.56–1.05)	0.77 (0.56–1.07)	0.116
Household crowding^c^										
No	1298.3	80	62	3	2	83	64	1.00	1.00	
Yes	2587	255	99	14	5	269	104	1.62 (1.21–2.17)^d^	1.45 (1.07–1.95)^d^	0.015
Toilet type										
Own flush	3527.5	303	86	16	5	319	90	1.00	1.00	
Shared flush	493.7	37	75	1	2	38	77	0.85 (0.57–1.28)	0.91 (0.58–1.41)	0.659
Other	18.6	3	161	1	54	4	215	2.37 (0.55–10.27)	1.81 (0.42–7.76)	0.423
Water source										
Piped to home	2711.8	251	93	13	5	264	97	1.38 (1.04–1.85)^d^	1.87 (0.81–4.34)	0.14
Bottled	1223.4	82	67	4	3	86	70	1.00	1.00	
Other	104.6	10	96	1	10	11	105	1.49 (0.68–3.27)	2.14 (0.70–6.53)	0.179
Water treatment										
Boil	2518.4	214	85	9	4	223	89	1.00	1.00	
Filter	269	41	152	4	15	45	167	1.88 (1.22–2.90)^d^	1.81 (1.17–2.81)^d^	0.008
None/other	1252.4	88	70	5	4	93	74	0.84 (0.63–1.12)	1.43 (0.62–3.27)	0.402

IYO, infant-years of observation; CI, cumulative incidence per 1000 infant-years; IRR, incidence rate ratio.

a <2500 g.

b One or more doses.

c Two or more people per room.

d Significant at *p* = <0.05.

**Table 4:** Risk factors for diarrhoeal presentation in Dong Thap

Characteristic	IYO	Outpatient		Hospitalized		Total diarrhoea		Unadjusted IRR	Adjusted IRR	*p*-Value
		Cases	CI	Cases	CI	Cases	CI			
Infant-years of follow-up	2199.4	1203	547	126	57	1329	604			
Maternal										
Age at delivery	-	-	-	-	-	-	-	0.98 (0.97–0.99)^c^	0.98 (0.96–0.99)^c^	0.006
Education										
Lower secondary or below	1693.8	1002	592	106	63	1108	654	1.00	1.00	
Higher secondary or above	503.4	200	397	19	38	219	435	0.67 (0.56–0.79)^c^	0.75 (0.62–0.91)^c^	0.004
Infant										
Sex										
Female	1077.9	564	523	45	42	609	565	1.00	1.00	
Male	1121.5	639	570	81	72	720	642	1.13 (0.99–1.3)^c^	1.20 (1.04–1.40)^c^	0.014
Low birth weight^a^										
No	2098.8	1150	548	123	59	1273	607	1.00	1.00	
Yes	100.6	53	527	3	30	56	557	0.92 (0.66–1.29)	0.91 (0.63–1.31)	0.603
Breastfed after birth?										
Yes, exclusively	1630.9	891	546	99	61	990	607	1.00	1.00	
Yes, plus formula	555.8	302	543	27	49	329	592	0.98 (0.84–1.15)	1.05 (0.88–1.26)	0.546
No, only formula	12.7	10	790	0	0	10	790	1.27 (0.56–2.89)	1.44 (0.60–3.44)	0.411
Older sibling										
No	1168.9	614	525	70	60	684	585	1.00	1.00	
Yes	1028.3	588	572	55	53	643	625	1.07 (0.93–1.22)	1.17 (0.98–1.39)	0.074
Household										
Household crowding^b^										
No	445	233	524	18	40	251	564	1.00	1.00	
Yes	1523.9	817	536	87	57	904	593	1.05 (0.88–1.26)	0.99 (0.82–1.19)	0.926
Toilet type										
Flush	1315.2	658	500	71	54	729	554	1.00	1.00	
None/bush	524	332	634	30	57	362	691	1.24 (1.05–1.46)^c^	1.03 (0.84–1.27)	0.782
Other	357.1	211	591	24	67	235	658	1.19 (0.98–1.43)	1.00 (0.81–1.24)	0.993
Water source										
Piped to home	682.1	345	506	32	47	377	553	1.08 (0.81–1.44)	2.10 (0.68–6.47)	0.198
Piped to public tap	246	119	484	10	41	129	524	1.02 (0.73–1.43)	1.53 (0.49–4.76)	0.463
Bottled	168.2	77	458	9	54	86	511	1.00	1.00	
River/stream	1026.9	613	597	67	65	680	662	1.29 (0.98–1.7)	2.28 (0.74–7.07)	0.153
Other	73	48	658	7	96	55	754	1.46 (0.95–2.26)	2.64 (0.83–8.36)	0.100
Water treatment										
Boil	1499.5	868	579	82	55	950	634	1.00	1.00	
Filter	508.8	237	466	33	65	270	531	0.84 (0.71–0.99)^c^	0.92 (0.76–1.13)	0.423
None	171.5	82	478	10	58	92	537	0.85 (0.65–1.11)	1.85 (0.61–5.57)	0.277
Other	16.5	15	910	0	0	15	910	1.44 (0.71–2.93)	1.56 (0.74–3.25)	0.250
Pigs in household										
No	1972.8	1058	536	113	57	1171	594	1.00	1.00	
Yes	224.4	144	642	12	53	156	695	1.17 (0.94–1.46)	1.06 (0.82–1.36)	0.633
Poultry in household										
No	1498.7	787	525	90	60	877	585	1.00	1.00	
Yes	698.4	415	594	35	50	450	644	1.11 (0.96–1.28)	0.97 (0.82–1.15)	0.710

IYO, infant-years of observation; CI, cumulative incidence per 1000 infant-years; IRR, incidence rate ratio.

a <2500 g.

b Two or more people per room.

c Significant at *p* = <0.05.
